# Effectiveness and safety of inpatient versus extended venous thromboembolism (VTE) prophylaxis with heparin following major pelvic surgery for malignancy: protocol for a systematic review

**DOI:** 10.1186/s13643-019-1179-1

**Published:** 2019-10-30

**Authors:** Bridget Heijkoop, Natalie Parker, George Kiroff, Daniel Spernat

**Affiliations:** Urology Department, The Queen Elizabeth Hospital, The University of Adelaide, Level 7A, 28 Woodville rd, Woodville South, SA 5011 Australia

**Keywords:** Venous thromboembolism, Prophylaxis, Postoperative, Heparin, Pelvic surgery, Malignancy, Safety

## Abstract

**Background:**

Venous thromboembolism (VTE) is a common postoperative complication associated with significant morbidity and mortality. The use of prophylactic heparin postoperatively reduces this risk, and the use of extended duration prophylaxis is becoming increasingly common. Malignancy and pelvic surgery both independently further increase the risk of postoperative VTE and patients undergoing major pelvic surgery for malignancy are at particularly high risk of VTE. However, the optimum duration of prophylaxis specifically in this population currently remains unclear.

**Methods:**

We will conduct a systematic review of literature in accordance with the Cochrane Handbook for Systematic Reviews of Interventions (Higgins JPT, Green S. Cochrane Handbook for Systematic Reviews of Interventions version 5.1.0.,2011) to evaluate current evidence of the effectiveness and safety of inpatient versus extended VTE prophylaxis with heparin (all forms) following major pelvic surgery for malignancy. We will search PubMed, EMBASE, and the Cochrane Library. Regarding safety, Food and Drug Administration (FDA), and Therapeutic Goods Administration (TGA) websites will be searched, including all levels of evidence. Results will be the postoperative timeframe in which a VTE event can be considered to have been provoked by the surgery, and the number of patients needed to treat with both inpatient and extended prophylaxis to prevent a VTE event in this timeframe, comparing these to determine if there is a significant benefit from extended prophylaxis.

**Discussion:**

This systematic review will aim to identify the postoperative period in which patients undergoing major pelvic surgery for malignancy are at further increased risk of VTE as a result of their surgery and the optimum duration of heparin VTE prophylaxis with heparin to reduce this risk. Determining this will allow evidence-based recommendations to be made for the optimum duration of heparin VTE prophylaxis post major pelvic surgery for malignancy, leading to improved standards of care that are consistent between different providers and institutions.

**Systematic review registration:**

In accordance with guidelines, our systematic review was submitted to PROSPERO for consideration of registration on 16/12/17 and was registered on 12/1/18 with the registration number CRD42018068961, and it was last updated on December 1, 2018.

## Background

Venous thromboembolism (VTE) is a common postoperative complication associated with significant morbidity and mortality [1]. A major risk factor for VTE is a type of surgery, with patients undergoing major oncological surgery or pelvic surgery being at a significant risk [1]. These patients frequently also have additional non-modifiable risk factors for VTE including advanced age, limited mobility, previous VTE, or hereditary pro-thrombotic disorders. However, these risks can be mitigated by providing mechanical and or pharmacological prophylaxis. Best practice guidelines including the current BJUI [2] recommendation and those previously produced by American Urological Association [1] recommend the use of low molecular weight (enoxaparin) or unfractionated heparin in patients who are at high risk of VTE.

However, despite the consensus that the risk of VTE extends for a significant period postoperatively, to date, literature reviews have found insufficient evidence to determine an exact timeframe for this and consequently have not been able to make an evidence-based recommendation for the optimum duration of prophylaxis [2]. In addition, there does not appear to be a consistent pattern of use of postoperative pharmacological VTE prophylaxis in pelvic oncological surgery patients.

As with all interventions, the benefit must be weighed against the potential for adverse events. Known complications of pharmacological DVT/VTE prophylaxis include both major and minor haemorrhage, thrombocytopaenia, elevation of serum aminotransferases, infection associated with haematoma, hypersensitivity reactions, and local reactions [3]. With an increased duration of prophylaxis, there will be an increase in prophylaxis related adverse events, up to a point where these outweigh any ongoing benefit of the prophylaxis—again, at what duration of prophylaxis this point is reached remains unclear.

Consequently, further investigation is warranted and we aim to define the optimum duration of postoperative pharmacological VTE prophylaxis with heparin following major pelvic oncological surgery to define the most effective reduction in population risk of VTE without disproportionately increasing the risk of heparin-associated complications. Identifying this and making an evidence-based recommendation would enable all pelvic oncological surgery patients to receive standardised best practice postoperative pharmacological VTE prophylaxis.

## Methods/design

### Aims

The aim of this systematic review is to review the currently available literature to evaluate the effectiveness and safety of inpatient versus extended VTE prophylaxis with heparin (all forms) following major pelvic surgery for malignancy answering the following questions:

1. Timeframe postoperatively in which a VTE event can reasonably be considered to have been provoked by the surgical procedure.
2. Number needed to treat (NNT) with inpatient prophylaxis to prevent one VTE event within the timeframe established by 1.
3. NNT with extended prophylaxis to prevent one VTE event within the timeframe established by 1.
4. Considering the results of 2 and 3, is there a significant benefit associated with extended prophylaxis in reducing the risk of VTE events?
5. Considering the result of 4, if extended prophylaxis is shown to significantly reduce VTE events in the postoperative period established in 1, does this benefit outweigh the associated risks of extended prophylaxis—i.e., is extended prophylaxis safe?

### Design—search strategy and information sources

Literature search regarding both effectiveness and safety will be conducted by searching PubMed, EMBASE (2008-present), and Cochrane databases from inception to present. Regarding safety, Food and Drug Administration (FDA) and Therapeutic Goods Administration (TGA) websites will be reviewed in addition to targeted searches of Health Technology Assessment databases such as ‘EuroSCAN’ [4] to identify any further grey literature reporting of adverse events that may have been reported to these bodies but not published within a research paper and therefore not captured by the database search.

In addition to the electronic database and regulatory website search described above, we will review public registries of clinical trials and the reference lists of included literature and the published work of authors listed, with the aid of tools such as SCOPUS.

Search terms will include the medical subject headings (MeSH) and keywords combined by Boolean operators: Venous Thromboembolism OR VTE OR Deep vein thrombosis OR DVT AND Pelvic OR Malignancy OR Oncology AND Heparin OR Enoxaparin AND Prophylaxis. Refer to Additional file 1 for search strategy.

### Design—eligibility criteria

1. We will include English language studies of NHMRC level of evidences I–V. The decision to include case reports (level V) and case series was made as it was felt these would be useful to identify adverse events.
2. Participants; participants will be the subjects of all included literature, including adult (18 years and older) patients of either gender undergoing pelvic surgery for malignancy.
3. The intervention will be the use of extended pharmacological VTE prophylaxis (as opposed to in hospital pharmacological prophylaxis) with any form of heparin postoperatively, with the assumption made that all patients receiving pharmacological prophylaxis also received non-pharmacological VTE prophylaxis (e.g., compression stockings, sequential compression devices).
4. The comparator will be inpatient versus extended use of heparin VTE prophylaxis.
5. Outcomes; endpoints considered will include the following:
  - VTE events
  - Heparin-associated complications; classified by Clavien-Dindo grade [5]
6. Studies published within the last 10 years will be included. The decision to exclude studies published more than 10 years ago was made to ensure that included literature more closely reflects recent clinical practice and is therefore relatively representative of the current day patient population.
7. Setting will be inpatient and community patients receiving pharmacological VTE prophylaxis with heparin postoperatively.

### Design—selection process

Retrieved articles will be independently reviewed by the primary author and peer reviewer and included or excluded by the pre-determined criteria described above.

Articles will first be shortlisted for inclusion or discarded based on their titles. Of those shortlisted by title, the abstract will be reviewed and the papers returned to the shortlist or discarded by the relevance of the abstract. Those included or unclear based on the abstract will proceed to review the entire article by both the primary and second author, who will independently document if they would include or discard the article.

Should there be a disagreement between the primary and secondary reviewers, the article will be additionally reviewed by the supervisors of the project and a reasonable effort made to contact the author for any clarification required on the included material.

Exclusion criteria will include non-English language papers as the team lacks the resources available to translate these, paediatric populations and animal studies. Non-English language papers with an English abstract will be listed as potentially relevant studies awaiting assessment in the review to alert the reader of these papers’ existence in a wider evidence base.

### Design—data collection process

A template form of data variables to be extracted will be produced, and reviewers will independently complete this for each article reviewed, and additionally transcribe the data points into separate excel spreadsheets. Each reviewer’s assessment will then be compared, and in the event of any discrepancies not able to be resolved on re-review, the article will be reviewed by a supervisor, following which if the discrepancies remain unresolved, reasonable attempts will be made to seek clarification from the author.

A template of this in a table form is included as Additional file 2*.*

### Design—data items

#### Variables to source data for

- Timing of starting heparin postoperatively.
- Total duration heparin prophylaxis; inpatient versus extended. This will be considered a dichotomous variable, with patients categorised into those receiving prophylaxis while a hospital inpatient or those receiving ongoing prophylaxis of any duration on discharge.
- VTE events.
- Event that may be considered complications of prophylaxis and determine their Clavien-Dindo grade
- Type of surgery (primary or revision). If sufficient data is identified, this may subsequently be used to conduct a further analysis comparing outcomes of primary and revision procedures.
- Disease histology. If sufficient data is identified, this may subsequently be used to conduct further analysis by each oncologic pathology.

#### Assumptions made

- Inpatient stay will be considered a single duration despite the fact that inpatient stay postoperatively may be of variable length as this remains of significant impact due to altered mobility/activity from baseline while in inpatient setting.
- Concurrent use of mechanical prophylaxis; it will be assumed all patient’s received mechanical VTE prophylaxis in addition to pharmacological as the instances in which this is contraindicated are uncommon.
- Assume all patients receive an appropriate dose of pharmacological prophylaxis for their individual condition (i.e., appropriate for body habitus, appropriate reduction in dose for impaired renal function). However, if it is possible from the included data to confirm an appropriate dose was used, this will be confirmed.
- Potential VTE events not diagnosed on ultrasound (US)/computed tomography pulmonary angiography (CTPA)/V/Q scan are of sufficiently insignificant impact on the individual’s recovery as to be irrelevant to the outcomes of the study.

### Design—outcomes prioritisation

The primary outcome will be the number of clinically evident VTE events in patients treated with prophylactic heparin following major pelvic surgery for malignancy, subdivided into those treated only while inpatient immediately postoperatively and those treated with an extended course. Clinically evident VTE will be defined as that confirmed on investigation with US/CTPA. Any incidence of VTE not detected by these means will be interpreted as being sufficiently minor as to be clinically insignificant. In addition, any diagnoses of VTE made purely on the basis of history and examination findings without objective evidence of confirmed VTE on these modalities will be excluded as the clinical presentation of VTE is nonspecific, and thus, this may not represent a true VTE event.

Secondary outcomes will include both adverse events attributable to the use of pharmacological VTE prophylaxis such as bleeding/haematoma/thrombocytopaenia/drug reaction and in association with identified VTE events; length of stay/ICU admission/readmission to hospital following discharge.

### Design—risk of bias and planned assessment of meta bias

Potential sources of bias in this review will be from pre-existing bias in reviewed articles, publication bias, and potential of data censuring. The risk will be minimised by the use of appropriate critical appraisal tools (e.g., AMSTAR2 for systematic reviews [6] and Cochrane tool [7] for randomised controlled trials) to critically assess each article and consider exclusion of low scoring articles.

### Design—data synthesis

Meta-analysis will be the preferred form of data analysis. Revman [8] software will be used to directly compare appropriate articles and produce graphical representations of the risk ratio using Mantel-Haenszel analysis, random effect model, and a 95% confidence interval. Statistical support will be sought to assist with this process.

### Design—how the strength of the body of evidence will be assessed

The strength of the body of evidence will be assessed using the GRADEpro tool [9].

### Design—study records

The record of the details (title, author(s), where published, date of publication, access date, reasoning behind decision to include) of all included articles will be kept in an excel spreadsheet. Those relevant to effectiveness will be kept on a separate page of the spreadsheet to those relevant to safety and those relevant to both effectiveness and safety duplicated across both pages.

The second author will keep an additional database of the articles they have reviewed and their reasoning for their recommendation that the article be included or discarded.

Details of articles initially identified on scoping search and subsequently excluded will be kept in a separate excel document which will also lists the reason for their exclusion.

### Design—process of dealing with amendments to protocol

A copy of the protocol will be saved and kept both without and with amendments, giving a new version number to the amended copy; so versions at all stages of amendments remain available. Other contributors will be notified of amendment via email.

## Discussion

As this protocol is for a systematic review of pre-existing literature, minimal operational issues are anticipated, with the primary difficulty anticipated being any situation where the full text of an identified article is not accessible. This will be managed on a case by case basis with the available text (e.g., abstract) of the article reviewed by both reviewers and the project supervisors to determine if it is suitable for inclusion or not. Should a consensus not be able to be reached the article will be included.

We acknowledge limitations of this review including potential sources of bias described in the ‘Methods/design’ section and including only English language publications.

## Supplementary information

**Additional file 1.** Search Strategy.

**Additional file 2.** Data Collection Template.

## Data Availability

Data analysed during the study will be available from the corresponding author on request.

## Abbreviations

AUA: American Urological Association
BJUI: British Journal of Urology International
DVT: Deep vein thrombosis
MeSH: Medical subject headings
NNT: Number needed to treat
TGA: Therapeutic Goods Administration
VTE: Venous thromboembolism
