# Supplementary material for: Effectiveness and safety of inpatient versus extended venous thromboembolism (VTE) prophylaxis with heparin following major pelvic surgery for malignancy: protocol for a systematic review
Source: Syst Rev. 2019 Oct 30;8:249. doi: 10.1186/s13643-019-1179-1 (PMC6822405; doi:10.1186/s13643-019-1179-1)

Additional File 1 – Search Strategy

Used for PubMed

(((((((((aspirin) OR dalteparin) OR warfarin) OR low molecular weight heparin) OR enoxaparin) OR heparin)) OR ((((((heparin, low molecular weigh[MeSH Terms]) OR aspirin[MeSH Terms]) OR warfarin[MeSH Terms]) OR dalteparin[MeSH Terms]) OR enoxaparin[MeSH Terms]))) AND (((((((((((((((((((cancer, uterine cervical[MeSH Terms] OR cerval cancer) OR urethral cancer[MeSH Terms]) OR urethral cancer) OR cancer, prostate[MeSH Terms]) OR prostate cancer) OR cancer, uterine[MeSH Terms]) OR uterine cancer) OR cancer, ovarian[MeSH Terms] OR ovarian cancer) OR colorectal cancer[MeSH Terms]) OR bowel cancer) OR cancer of the ureter[MeSH Terms]) OR ureteric cancer) OR bladder cancer) OR bladder cancer[MeSH Terms])) OR ((((“Abdominal Neoplasm”[Mesh]) OR abdominal neoplasm)) OR ((pelvic neoplasms) OR “Pelvic Neoplasms” [Mesh])))


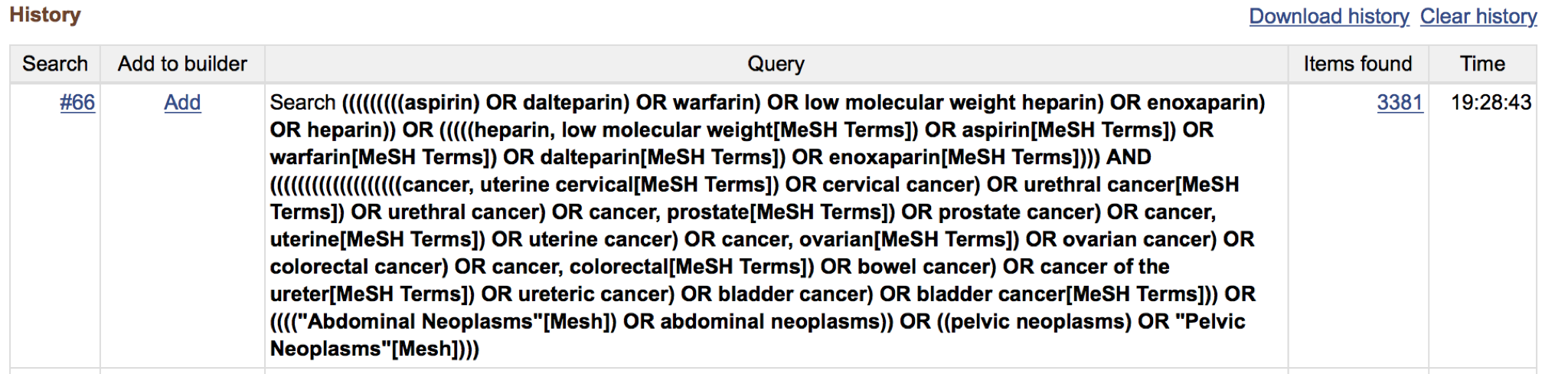

Supplement: Supplementary file 1 — Additional file 1. Search Strategy. [file 13643_2019_1179_MOESM1_ESM.docx]
