# Supplementary material for: Effectiveness and safety of inpatient versus extended venous thromboembolism (VTE) prophylaxis with heparin following major pelvic surgery for malignancy: protocol for a systematic review
Source: Syst Rev. 2019 Oct 30;8:249. doi: 10.1186/s13643-019-1179-1 (PMC6822405; doi:10.1186/s13643-019-1179-1)
Supplement: Supplementary file 2 — Additional file 2. Data Collection Template. [file 13643_2019_1179_MOESM2_ESM.docx]

ADDITIONAL FILE 2: DATA COLLECTION TEMPLATE

| Article | 1 | 2 | 3 |
| --- | --- | --- | --- |
| Title |  |  |  |
| Author(s) |  |  |  |
| Year of Publication |  |  |  |
| Journal |  |  |  |
| NHMRC Evidence Level |  |  |  |
| Malignancy diagnosis/ese |  |  |  |
| Procedure(s) |  |  |  |
| Major intervention  (Including dose/route/frequency/duration)  - Number post exclusions |  |  |  |
| Comparator  (Including dose/route/frequency/duration)  - Number post exclusion |  |  |  |
| Outcomes  - How were these defined  - How were these measured |  |  |  |
| Conclusions |  |  |  |
| EFFICACY OUTCOMES | | | |
| Mortality rate secondary VTE |  |  |  |
| Rate of VTE  - Symptomatic  - Asymptomatic  - Total |  |  |  |
| DVT:PE |  |  |  |
| SAFETY OUTCOMES | | | |
| Bleeding events  - Major  - Minor  - Total |  |  |  |
| Number of transfusions |  |  |  |
| Wound complications |  |  |  |
| Other adverse events |  |  |  |
| Readmissions |  |  |  |
| Estimated intraoperative blood loss |  |  |  |
